# Effect of heptavalent pneumococcal conjugate vaccination on invasive pneumococcal disease in preterm born infants

**DOI:** 10.1186/1471-2334-10-12

**Published:** 2010-01-19

**Authors:** Simon Rückinger, Mark van der Linden, Rüdiger von Kries

**Affiliations:** 1 Ludwig-Maximilians-University of Munich, Institute of Social Pediatrics and Adolescent Medicine, Division of Epidemiology, Germany; 2 National Reference Center for Streptococci, Institute of Medical Microbiology, University Hospital RWTH Aachen, Germany

## Abstract

**Background:**

Evidence for protection of preterm born infants from invasive pneumococcal disease (IPD) by 7-valent pneumococcal conjugate vaccination (PCV7) is relatively sparse. Data from randomized trials is based on relatively small numbers of preterm born children.

**Methods:**

We report data from active prospective surveillance of IPD in children in Germany. The cohorts of preterm born children in 2000 and 2007 and the respective whole birth cohorts are compared regarding occurrence of IPD.

**Results:**

After introduction of PCV7 we observed a reduction in the rate of IPD in preterm born infants comparing the 2000 and 2007 birth cohort. The rate of IPD among the whole birth cohorts was reduced from 15.0 to 8.5 notifications per 100,000 (*P *< .001). The impact among the preterm birth cohort was comparable: A reduction in notification rate from 26.1 to 16.7 per 100,000 comparing the 2000 with the 2007 preterm birth cohort (*P *= .39). Preterm born infants with IPD were either unvaccinated or vaccinated delayed or incomplete.

**Conclusions:**

This adds to evidence that PCV7 also protects preterm born infants effectively from IPD. Preterm born infants should receive pneumococcal vaccination according to their chronological age.

## Background

*Streptococcus pneumoniae *is a major source of morbidity and mortality worldwide. It is estimated that *S. pneumoniae *causes around 11% of all deaths in children aged 1-59 months [1]. Invasive pneumococcal disease (IPD) in children is associated with considerable case-fatality rates and rates of sequelae, even in industrialized countries [2-6]. In February 2000, a 7-valent pneumococcal conjugate vaccine (PCV7) was licensed in the US. This vaccine offers the possibility for a general vaccination program in infants and toddlers. Starting from 2001, a strong decrease of IPD incidence in children has been reported from the US [7-11]. Several European countries have shown decreases in the incidence of IPD after introduction of PCV7 [12] including Germany [13].

Preterm born infants are at higher risk for infectious diseases like invasive pneumococcal disease (IPD), with the highest risk in early infancy [14]. Vaccination of preterm born infants is often performed delayed [15]. Efficacy of heptavalent pneumococcal conjugate vaccination (PCV7) to protect infants younger than 2 years from IPD was shown in a randomized controlled trial [16]. Evidence from this trial also suggested a good protection of preterm born infants [17]. A study on immunogenicity of PCV7 from Italy in 46 preterm born infants showed antibody levels comparable to those in 46 full term infants [18]. However, another immunogenicity study from the UK with 68 preterm infants and 69 full term infants showed lower antibody levels among the preterm infants [19]. Furthermore, the lack of evidence of protection by PCV7 of preterm infants born before 32 weeks gestation from IPD has been stressed [20]. General vaccination with PCV7 of all infants was recommended in July 2006 in Germany. In this study we evaluate the impact of PCV7 on IPD in preterm born infants in Germany comparing children born in 2000 with those born in 2007.

## Methods

We report data from active prospective surveillance of IPD in all German pediatric hospitals. Hospitals were contacted on a monthly basis via postcards to ask if a case of invasive pneumococcal disease had been observed the last month. The case definition of IPD was isolation of *Streptococcus pneumoniae *from at least one culture of blood, cerebrospinal fluid, or a sample from any other normally sterile body site. Cases were excluded if they did not match the case definition, were older than 15 years, or double reports. Physicians filled comprehensive questionnaires on disease manifestation, outcome, vaccinations, and underlying conditions. There was a check box specifically asking for preterm birth and week of gestation of preterm births. Details of the study have been described elsewhere [13,21,22]. The study is part of the International Network of Paediatric Surveillance Units (INoPSU) [23].

Microbiologic laboratories were invited to send pneumococcal strains isolated from a normally sterile body site to the German National Reference Center for Streptococci, where the species identification was confirmed and serotyping, using Neufeld's Quellung method [24], was performed.

In order to account for a population denominator, the number of preterm born children (before 37 completed weeks gestation) was estimated based on the assumption that about 7% of children in Germany are born preterm. Based on the hospital surveillance, we compared data from two preterm birth cohorts: those born in 2000 and those born in 2007. Infants from both birth cohorts were under surveillance for at least one and two years at maximum: 2000-2001 and 2007-2008 respectively. Also based on the hospital surveillance we compared IPD notifications for all children (irrespective whether preterm born or not) for the birth cohorts of the years 2000 and 2007 using the same approach. Numbers on all births in Germany were provided by the Federal Statistical Office Germany. Incidence estimates were calculated based on IPD notifications from the respective (preterm) birth cohort reported in the year of birth and the subsequent year. These total numbers were divided by the respective number of (preterm) born children. Incidence estimates from 2000-2001 and 2007-2008 were compared using Fisher's exact test for difference in proportions.

Because this study is based on retrospective case analysis, without the possibility of identifying individual subjects, only data protection issues are of concern. Consent of the Bavarian data protection office was given.

## Results

Between January 2007 and December 2008, 58 cases of IPD in children born in 2007 were reported from Germany pediatric hospitals compared to 115 cases in children born in 2000 reported between 2000 and 2001 (Table 1). This corresponds to a reduction in notifications per 100,000 from 15.0 to 8.5 (*P *< .001). The reported number of preterm born children with IPD was 14 in 2000-2001 and 8 in 2007-2008. This corresponds to a reduction in notification rate from 26.1 to 16.7 per 100,000 comparing the 2000 with the 2007 preterm birth cohort (*P *= .39). Among the 14 cases of IPD among preterm born children, 9 had information on serotype and for 7 of these the serotype was included in PCV7 (14 (3 cases), 18C (2 cases), 19A, 23F, 3, 9V).

**Table 1:** Births and preterm births in 2000 and 2007 in Germany and invasive pneumococcal disease (IPD) cases reported from these birth cohorts in 2000-2001 and 2007-2008 respectively.

	Birth cohort			
			
	2000	2007	Percentage decrease	*P*^a^
All infants:				
Births	766,999	684,862		
IPD notifications	115	58		
IPD notifications per 100,000	15.0	8.5	43.3	< .001
Preterm born infants:				
Births^b^	53,690	47,940		
IPD notifications	14	8		
IPD notifications per 100,000	26.1	16.7	36.0	.39

^a ^*P *value calculated with Fisher's exact test.

^b ^Estimated based on a prevalence of preterm birth of 7%.

Among the preterm born children born in 2000, 6 out of 14 had a gestational age of less than 32 weeks and one child had bronchopulmonary dysplasia. Among the preterm born children born in 2007, 3 out of 8 had a gestational age of less than 32 weeks and one child had partial trisomy 15.

Characteristics of the eight IPD cases in children born preterm in 2007 reported between 2007 and 2008 are shown in Table 2. Five out of eight cases were born after the 32^nd ^week gestation. Four cases were vaccinated with PCV7 prior to disease onset. However no infant had received full vaccination with booster dose according to the recommended scheme. In two cases a serotype included in PCV7 was found. These two cases had not received pneumococcal vaccination prior to disease onset, although they were at an appropriate age. In two cases a neurological deficit was reported as outcome and in one case a cerebral parenchymal damage.

**Table 2:** Characteristics of invasive pneumococcal disease (IPD) of preterm births born in 2007 from surveillance 2007-2008.

ID	Sex	Gestational week	IPD at age (months)	PCV7 vaccination	Sequelae	Serotype
1	M	24	9	8 months	Neurological deficit	15B
2	F	30	8	-	Cerebral parenchymal damage	23Fa
3	M	31	22	4, 5, and 7 months	-	Unknown
4	F	35	20	-	Unclear	14a
5	F	36	9	3 and 4 months	-	10A
6	F	36	3	-	-	Unknown
7	M	36	6	-	-	Unknown
8	M	37	5	4 months	Neurological deficit	19A

^a ^Serotype included in PCV7.

## Discussion

Comparing the 2000 and 2007 birth cohorts we have observed a similar reduction in IPD notifications in preterm born children compared to children born at term after introduction of the general pneumoccal vaccination programme in Germany. These data suggest that PCV7 vaccination is similarly effective in protecting preterm born infants from IPD compared to full term children.

Our results add to previous evidence of protection of preterm born infants from IPD from a randomized controlled trial [17], and studies on immunogenicity of PCV7 in preterm infants [18,19]. In the randomized controlled trial 4,340 preterm born children were enrolled and none of these had IPD in the intervention group compared to 9 IPD cases in the control group. The about 2-fold higher IPD rates among preterm born children we observed are in line with the finding of a 1.6-fold increased risk of IPD in preterm born children from this clinical trial [17].

It is relatively unlikely that the reduction of IPD incidence among preterm born children was influenced by herd immunity. The vaccination program was not fully implemented before January 2007 and it was estimated that less than 80% of all children born in 2007 were fully vaccinated [13]. Therefore, it is most likely that these results rather reflect direct protection of the vaccine.

The distribution of gestational age among preterm born children with IPD from the 2007 preterm birth cohort does not suggest that infants born before 32 weeks gestation are protected considerably less than infants born after 32 weeks gestation. However, case numbers and therefore statistical power may be too small for a conclusive answer and future studies on the effectiveness of PCV7 in very preterm born infants are needed.

Timing of vaccination in preterm infants born in 2007 was not in accordance with the German guidelines that advocate vaccination at 2, 3, 4, and 11-14 months. Four cases had received no vaccination although they were at an appropriate age before disease onset. This confirms concerns about the tendency towards delayed immunization of preterm infants, exposing them for infection at time points where they are most vulnerable [15].

This study is somehow limited by the fact that the exact number of preterm born infants in Germany is unknown. There are complete surveys on births in some parts of Germany. Data from these surveys suggest that the proportion of preterm born infants was stable regarding the study period (personal communication by Dr. Lack, Bavarian Quality Assurance Institute for Medical Care, Munich, Germany). Another limitation is the lack of trend data, e.g. for the birth cohorts between 2001 and 2006. The hospital surveillance system was confined to vaccinated cases between 2003 and 2006. Furthermore, a recommendation to vaccinate preterm born children with PCV7 was issued in 2001. Therefore the 2000 birth cohort represents the only birth cohort for which data on unvaccinated preterm born children could be analysed.

## Conclusions

In summary this study adds to evidence that PCV7 also protects preterm born infants from IPD and points to the need for timely vaccination of preterm children according to their chronological age as recommended e.g. by the German Standing Vaccination Committee (STIKO) and the American Academy of Pediatrics Committee on Infectious Diseases [25].

## Competing interests

RvK and MvdL have received grant support from Wyeth

## Authors' contributions

SR suggested the idea for the article, performed the statistical analysis and wrote the manuscript. MvdL contributed to the writing of the manuscript and revised the final version for important intellectual content. RvK supervised the writing of the manuscript and revised the final version of the manuscript for important intellectual content.

## Pre-publication history

The pre-publication history for this paper can be accessed here:

http://www.biomedcentral.com/1471-2334/10/12/prepub
